# De Novo Pure Erythroid Leukemia With Rapid Progression and Multiple Lytic Bone Lesions: A Case Report

**DOI:** 10.7759/cureus.41581

**Published:** 2023-07-08

**Authors:** Shoichiro Okazaki

**Affiliations:** 1 Hematology, Ichinomiya Municipal Hospital, Ichinomiya, JPN

**Keywords:** azacytidine, acute myeloid leukemia, e-cadherin, tp53 mutation, multiple lytic bone lesions, pure erythroid leukemia

## Abstract

A 62-year-old male patient presented with malaise and severe macrocytic anemia. Computed tomography revealed an osteolytic lesion in the left iliac bone. Bone marrow examination revealed that 90% of erythroblasts were large with periodic acid-Schiff (PAS)-positive staining while flow cytometry and immunostaining revealed CD71 (+), GP-A (+), p53 (+), CD117 (+), and CD34 (−) results, indicating pure erythroid leukemia (PEL) diagnosis. A needle biopsy of the osteolytic lesion revealed the same characteristics as PEL. Azacitidine therapy was administered as the first-line treatment, and his general condition temporarily improved. However, PEL quickly deteriorated, and he died 42 days of hospitalization after initial admission. PEL is an extremely rare form of acute myeloid leukemia (AML) and has presented cytogenetic characteristics in addition to the TP53 mutation. Other AML treatment is used because a standard treatment method is unavailable. However, the prognosis is extremely poor. Furthermore, few cases of concurrent bone lesions are reported globally, and more cases must be accumulated and analyzed.

## Introduction

Acute myeloid leukemia (AML) is a highly diverse hematologic tumor characterized by clonal autonomous growth of myeloid cells with impaired differentiation and maturation. Pure erythroid leukemia (PEL) is a rare form of AML, accounting for <5% of all AML cases. PEL, classified as AML-not otherwise specified according to the World Health Organization Classification of Tumors of Hematopoietic and Lymphoid Tissues, 5th edition, is a disease characterized by the neoplastic proliferation of early erythroid precursors constituting >80% of nucleated bone marrow cells, without a significant myeloblast component. PEL frequently presents with complex karyotype and TP53 mutations while its etiology and molecular mechanisms remain unknown. As a consequence, there are currently no established treatments, and the prognosis for PEL is extremely poor [1].

Conversely, the involvement of extramedullary lesions during diagnosis in de novo AML is rare, occurring in only 1.1% of cases, and bone involvement accounts for 5%-16% of such cases [2]. Herein, we report a case of de novo PEL with rapid progression and multiple lytic bone lesions during diagnosis.

## Case presentation

**Figure 1:**
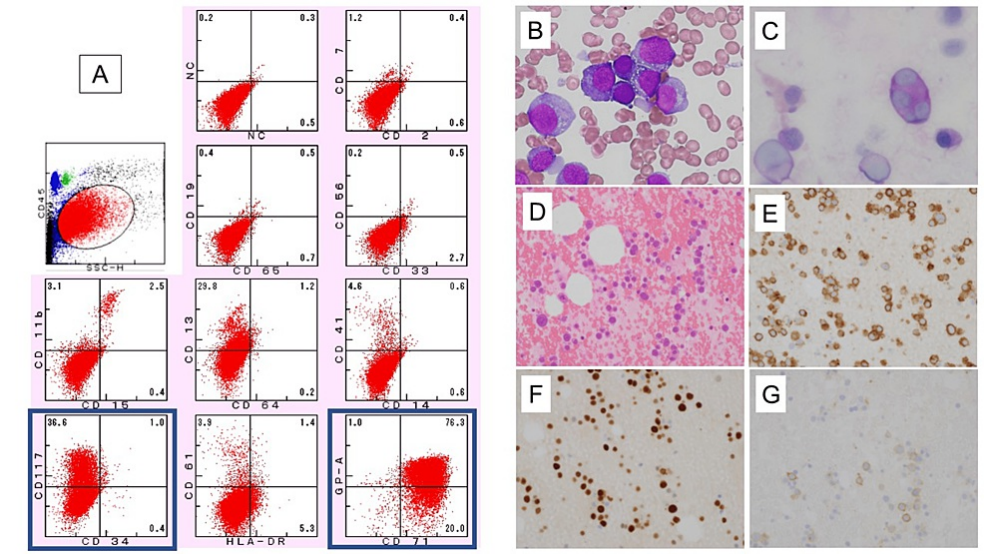
Abnormal erythroblasts in bone marrow findings A) Flow cytometry; B) May-Giemsa staining, ×1000; C) Periodic acid-Schiff staining, ×400; D) Hematoxylin and eosin staining, ×400; E) Immunostaining for CD71, ×400; F) Immunostaining for p53, ×400; G) Immunostaining for CD117, ×400

A 62-year-old male patient, with a history of megaloblastic anemia associated with alcoholism presented with low-grade fever and fatigue, was referred to our Department of Gastroenterology. Peripheral blood examination revealed macrocytic anemia and imaging studies showed lytic lesions in the left iliac, Th7, Th10, L5, and left rim bone. Upper gastrointestinal endoscopy revealed no abnormalities. Multiple myeloma was suspected, and a hematologist performed further evaluations. Laboratory tests revealed elevated serum lactate dehydrogenase (LDH) and C-reactive protein (CRP) levels and positive procalcitonin levels but with no coagulation abnormalities. Bone marrow examination revealed that 43% of atypical cells morphologically resembling myeloma cells with a nucleolus, and basophilic cytoplasm on smear showed expression of CD71 (+), CD117 (+), and CD34 (-) on flow cytometry. Myeloid blasts were not observed. Genetic analysis revealed negative chimeric screening, FLT3-ITD mutation, and JAK2 V617F mutation. G-banding karyotyping revealed a complex karyotype. Bone marrow pathological examination revealed hypercellular marrow with CD71 (+) and CD34 (-) erythroblasts expressing p53 (+) and CD117 (+). The PEL diagnosis was made based on histological features (Table 1, Figure 1).

**Table 1:** Laboratory findings at diagnosis WBC : White Blood Cell; Neutro : Neutrophil; Lymph : Lymphocyte; Atyp L : Atypical Lymphocyte; Eosino : Eosinophil; Baso : Basophil; Mono : Monocyte; RBC : Red Blood Cell; Hb : Hemoglobin; Hct : Hematocrit; MCV : Mean Corpuscular Volume; MCH : Mean Corpuscular Hemoglobin; MCHC : Mean Corpuscular Hemoglobin Concentration; Ret : Reticulocytes; Plt : Platelet; PT-INR : Prothrombin International Normalized Ratio; APTT : Activated Partial Thromboplastin Time; Fbg : Fibrinogen; FDP : Fibrin Degradation Products; WT1 : Wilms Tumor 1; ALP : Alkaline Phosphatase; AST : Asparate Aminotransferase; ALT : Alanine Aminotransferase; LDH : Lactate Dehydrogenase; γ-GTP : γ-Glutamyl Transpeptidase; Na : Sodium; K : Potassium; Cl : Chloride; Ca : Calcium; Fe : Serum Iron; Crea : Creatinine; BUN : Urea Nitrogen; UA : Uric Acid; GLU : Glucose; TP : Total Protein; Alb : Albumin; T-Bil : Total Bilirubin; CRP : C-Reactive Protein; PCT : Procalcitonin; IL-2R : Interleukin-2 Receptor; IgG : Immunoglobulin G; IgA : Immunoglobulin A; IgM : Immunoglobulin M; M/E Ratio : Myeloid/Erythroid Ratio

Complete Blood Count	
WBC	3200 /μL
Neutro	85%
Lymph	7%
Atyp. L	2%
Eosino	1%
Baso	0%
Mono	5%
RBC	1.83×10^6^ /μL
Hb	5.7 g/dL
Hct	16.50%
MCV	90.2 fL
MCH	31.1 pg
MCHC	34.5 g/dL
Ret	0.1%
Plt	10.9×10^4^ /μL
Coagulation	
PT-INR	1.12
APTT	35.9 sec
Fbg	564 mg/dL
FDP	3.4 μg/mL
RQ-PCR	
WT1	1500 copies/μgRNA
Blood Biochemistry	
ALP	127 U/L
AST	75 U/L
ALT	30 U/L
LDH	1455 U/L
γ-GTP	36 U/L
Na	129 mmol/L
K	3.9 mmol/L
Cl	94 mmol/L
Ca	9.1 mmol/L
Fe	26 μg/dL
Crea	2.77 mg/dL
BUN	30.8 mg/dL
UA	7.4 mg/dL
GLU	133 mg/dL
TP	6.1 g/dL
Alb	2.9 g/dL
T-Bil	0.66 mg/dL
CRP	22.53 mg/dL
PCT	(2+)
IL-2R	2490 U/mL
Serology	
IgG	964 mg/dL
IgA	260 mg/dL
IgM	45 mg/dL
Ferritin	758 ng/mL
Bone marrow	
Nucleated cell count	37000 /µl
M/E ratio	0.1
Myeloblast	0.00%
Proerythroblast	1.50%
Baso-erythroblast	17.00%
Poly-erythroblast	13.00%
Ortho-erythroblast	16.00%
Atypical cell	43.00%
Screening of chimeric genes related to leukemia	
Major BCR-ABL1, minor BCR-ABL1,	All negative
PML-RARA, RUNX1-RUNX1T1,	
CBFB-MYH11, DEK-NUP214,	
NUP98-HOXA9, ETV6-RUNX1,	
TCF3-PBX1, STIL-TAL1,	
KMT2A-AFF1, KMT2A-AFDN,	
KMT2A-MLLT3, KMT2A-MLLT1	
Chromosomal analysis	
A : 44, XY, -3, add (3) (p21), -5, -13, -15, -16, -17, add (19) (p13), +4mar {1}	
		B : 46, XY {1}	
【Complex karyotype derived from type A】	
43 : {1}, 44 : {12}, 45 : {3}, 88 : {1}, 94 : {1}	

**Figure 2:**
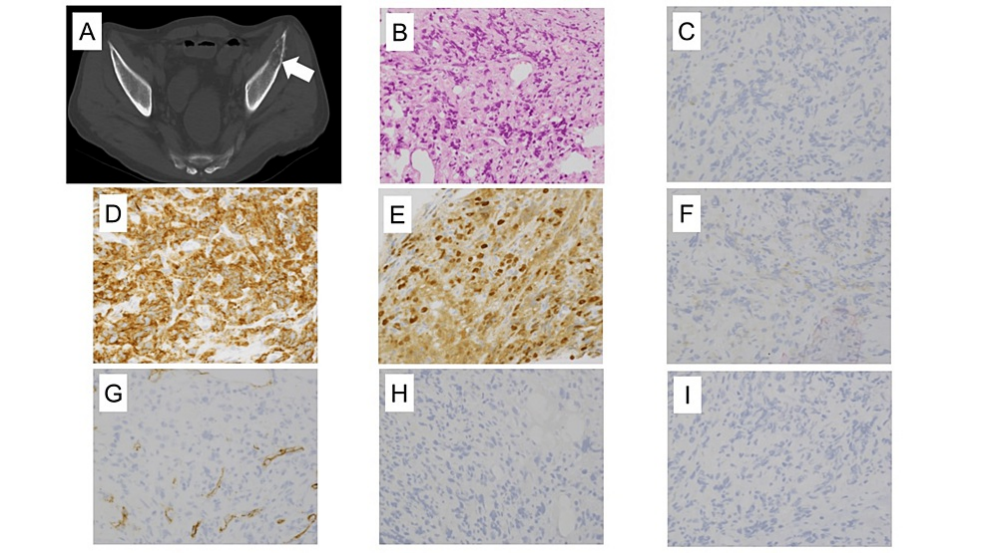
Initial imaging examination and pathological findings A) Dynamic abdominal computed tomography (CT): the biopsy site targeted within osteolytic lesion of left iliac bone (white arrow); B) Hematoxylin and eosin staining, ×400; C) Immunostaining for E-cadherin, ×400; D) Immunostaining for CD71, ×400; E) Immunostaining for p53, ×400; F) Immunostaining for CD117, ×400; G) Immunostaining for CD34, ×400; H) Immunostaining for CD61, ×400; I) Immunostaining for CD138, ×400

A computed tomography (CT)-guided needle biopsy was performed on a lytic lesion of the left ilium before the definitive PEL diagnosis, which revealed a large atypical cell proliferation on hematoxylin and eosin staining, and CD71(+), p53(+), and CD117(+) were detected on immunohistochemical staining, similar to those in bone marrow, confirming that it was a lytic lesion due to PEL. E-cadherin, which is positive in erythroblast precursor cells, was negative (Figure 2).

**Figure 3:**
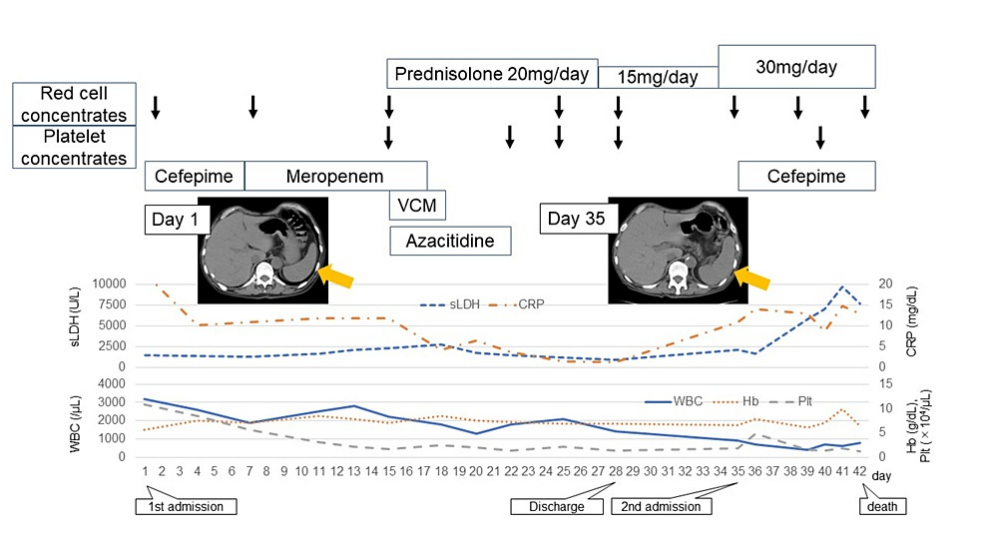
The clinical course of the present case VCM : Vancomycin; sLDH : Serum Lactate Dehydrogenase; CRP : C-Reactive Protein; WBC : White Blood Cell; Hb : Hemoglobin; Plt : Platelet

The patient showed signs of dehydration and hypotension and was diagnosed with sepsis upon admission. He received cefepime (CFPM) treatment and was hospitalized. The blood culture obtained on the day of admission was negative. The antibiotics were switched from CFPM to meropenem on the seventh day of hospitalization. Vancomycin was added on the fifteenth day because the fever could not be controlled, but his body temperature did not improve. Bone marrow examination and left ilium biopsy confirmed the PEL diagnosis, but his performance status (PS) was poor, and standard induction therapy for remission was determined to be difficult. Therefore, he was treated with azacytidine (AZA) monotherapy starting from the fifteenth day of hospitalization. Further, steroid therapy was initiated in parallel with AZA therapy, considering tumor fever, and his fever quickly improved, as evidenced by serum LDH level improvements on blood tests. He was discharged from the hospital on the 28th day of hospitalization. However, he had a fever and his serum LDH and CRP levels worsened again during his outpatient visit one week after discharge, leading to readmission. CT scans revealed worsening splenomegaly and lytic lesions compared to before AZA therapy. AZA monotherapy alone would be insufficient to control his condition; thus, we planned to switch to AZA + venetoclax (VEN) therapy. However, his serum LDH levels rose to 9654 U/mL due to the rapid PEL progression, and he developed disseminated intravascular coagulation. He died on the 42nd day of hospitalization after initial admission (Figure 3).

## Discussion

PEL has a very poor prognosis, with a median survival of 1.4-3 months [3]. PEL has no standard treatment, and young patients undergo induction therapy followed by consolidation therapy or allogeneic stem cell transplantation depending on prognostic factors, similar to other AML cases. In recent years, AZA monotherapy or AZA + VEN therapy has become an option for AML in elderly patients or those with difficult-to-treat AML [4-6]. AZA monotherapy was chosen in this case, as the initial treatment due to poor PS. Serum LDH, which was derived from ineffective PEL hematopoiesis demonstrated a tendency toward improvement, and overall condition also improved immediately after treatment initiation, allowing for temporary discharge. However, serum LDH rapidly increased before the start of the next treatment, and PEL could not be controlled.

A retrospective analysis of 41 cases of de novo PEL from Mayo Clinic, which used VEN along with hypomethylating agents as the initial treatment in 12 cases, revealed that all patients were treatment-resistant or died within one year of recurrence [7], and its effect on prognosis would have been limited even if AZA + VEN therapy had been implemented in this case.

PEL rarely presents with osteolytic lesions, and to the extent of our research, this case report is the second PEL case with bone involvement reported in the literature [8-11]. This case presented a diagnostic challenge, as the patient exhibited morphological similarities to plasmacytoma with undifferentiated erythroblasts and multiple lytic bone lesions, which raised multiple myeloma suspicion. CD71, which is a transferrin receptor protein, is strongly expressed in PEL but not lineage-specific. Glycophorin A (GPA) is specific to erythroblasts but may be negative in some PEL cases that are composed of early or immature erythroblasts. E-cadherin is a marker that becomes positive in such undifferentiated erythroblasts when GPA or CD71 is weakly positive or negative and may be a useful marker in the histopathological PEL diagnosis [12]. This case was positive for CD71 and p53 staining by immunohistochemistry and GPA by flow cytometry but negative for E-cadherin and so the diagnosis of PEL. Loss of the cell adhesion molecule E-cadherin has promoted bone metastasis and solid cancer invasion [13] and may be related to the multiple osteolytic lesions seen in this case. EF Reinig et al. reported the clinical pathological and cytogenetic features of 15 de novo PEL cases. Among the patients analyzed, only one case showed negative staining for E-cadherin in immunohistochemistry. Furthermore, this patient had the shortest interval from diagnosis to death, which was 0.2 months, among the reported cases of PEL [3].

## Conclusions

PEL with multiple lytic bone lesions has been rarely reported in the literature. Furthermore, it may be associated with organ dysfunction and poor prognosis. In cases of PEL diagnosis, comprehensive imaging studies and extensive workup are necessary. While E-cadherin is an important marker for diagnosing PEL, it was negative in this particular case. The loss of E-cadherin, distinct from typical PEL, may be involved in the development of multiple extramedullary lesions and treatment resistance, highlighting the need to accumulate more cases for further investigation.

## References

[1] Wang W, Wang SA, Medeiros LJ, Khoury JD (2017). Pure erythroid leukemia. Am J Hematol.

[2] Shallis RM, Gale RP, Lazarus HM (2021). Myeloid sarcoma, chloroma, or extramedullary acute myeloid leukemia tumor: a tale of misnomers, controversy and the unresolved. Blood Rev.

[3] Reinig EF, Greipp PT, Chiu A, Howard MT, Reichard KK (2018). De novo pure erythroid leukemia: refining the clinicopathologic and cytogenetic characteristics of a rare entity. Mod Pathol.

[4] Miyazaki Y (2020). Acute myeloid back leukemia. Japanese Society of Hematology. Guidelines for the Treatment of Hematopoietic Tumors (2018 Revised Edition).

[5] Erba HP (2015). Finding the optimal combination therapy for the treatment of newly diagnosed AML in older patients unfit for intensive therapy. Leuk Res.

[6] Pollyea DA, Pratz K, Letai A (2021). Venetoclax with azacitidine or decitabine in patients with newly diagnosed acute myeloid leukemia: Long term follow-up from a phase 1b study. Am J Hematol.

[7] Reichard KK, Tefferi A, Abdelmagid M, Orazi A, Alexandres C, Haack J, Greipp PT (2022). Pure (acute) erythroid leukemia: morphology, immunophenotype, cytogenetics, mutations, treatment details, and survival data among 41 Mayo Clinic cases. Blood Cancer J.

[8] Johnson JL, Moscinski L, Zuckerman K (2004). Value of positron emission tomography scan in staging cancers, and an unusual presentation of acute myeloid leukemia. Case 3. Acute myeloid leukemia presenting with lytic bone lesions. J Clin Oncol.

[9] Geetha N, Sreelesh KP, Priya MJ, Lali VS, Rekha N (2015). Osteolytic bone lesions - a rare presentation of AML M6. Mediterr J Hematol Infect Dis.

[10] Muler JH, Valdez R, Hayes C, Kaminski MS (2002). Acute megakaryocytic leukemia presenting as hypercalcemia with skeletal lytic lesions. Eur J Haematol.

[11] Dharmasena F, Wickham N, McHugh PJ, Catovsky D, Galton DA (1986). Osteolytic tumors in acute megakaryoblastic leukemia. Cancer.

[12] Caldwell I, Ruskova A, Royle G, Liang J, Bain BJ (2019). Pure erythroid leukemia: the value of E-cadherin in making the diagnosis. Am J Hematol.

[13] Onder TT, Gupta PB, Mani SA, Yang J, Lander ES, Weinberg RA (2008). Loss of E-cadherin promotes metastasis via multiple downstream transcriptional pathways. Cancer Res.

